# Grip Force Coordination as a Determinant of Independence in Activities of Daily Living and Instrumental Activities of Daily Living in Incomplete Cervical Spinal Cord Injury: A Case Report

**DOI:** 10.7759/cureus.106447

**Published:** 2026-04-04

**Authors:** Mitsuo Nakamura, Kotaro Kojima, Mariko Nakamura, Kiichi Sato, Satoko Matsumoto Harmon

**Affiliations:** 1 Department of Occupational Therapy, School of Health Sciences, Sapporo Medical University, Sapporo, JPN; 2 Department of Rehabilitation Medicine, Hokkaido Spinal Cord Injury Center, Bibai, JPN; 3 Department of Occupational Therapy, Sapporo Medical University, Sapporo, JPN

**Keywords:** adl (activities of daily living), cervical spinal cord injury, grip force modulation, hand dexterity, tenodesis grasp

## Abstract

Upper limb rehabilitation for individuals with cervical spinal cord injury (CSCI) primarily emphasizes strengthening residual muscles to enable grasping through the tenodesis action. While this approach is essential for achieving basic activities of daily living (ADLs), smooth object manipulation and fine motor performance require not only muscle strength but also the ability to regulate grip force. However, grip force modulation during tenodesis-based grasping has rarely been evaluated or specifically targeted in rehabilitation for cervical SCI. We report the case of a right-handed man in his late 50s with a C4-level cervical SCI who aimed to regain independence in daily activities and return to work as a school teacher. Although conventional occupational therapy led to improvements in upper limb strength and basic ADL, he continued to experience difficulty performing fine motor tasks, particularly handwriting, which required precise grip force control. Grip force regulation was quantitatively evaluated using a visual feedback-based force adjustment task, revealing greater errors during force-increasing phases than during force-decreasing phases. Based on these findings, occupational therapy was modified to incorporate training focused on grip force modulation using wrist extension, combined with explicit feedback and task-oriented practice in daily activities. At discharge, upper limb motor scores showed no further improvement; however, ADL performance improved, as reflected by higher Spinal Cord Independence Measure III scores, and the patient successfully returned to work, including performing handwriting tasks. This case suggests that assessing and training grip force regulation, in addition to muscle strengthening, may enhance functional upper limb use.

## Introduction

Upper-limb and hand dysfunction in individuals with cervical spinal cord injury (CSCI) significantly limits activities of daily living (ADLs). Therefore, occupational therapy focuses on maximizing residual function to promote independence in ADL [1-4]. In individuals with CSCI who lack sufficient finger flexor strength, grasping can be achieved without active finger flexion by utilizing the tenodesis action produced by wrist extension [5]. This function is critically important for the acquisition of ADL, and interventions primarily focusing on strengthening the muscles of the upper limb and fingers have enabled grasping and manipulation of objects used in daily activities [6].

However, smooth manipulation of objects in daily life and the performance of tasks requiring higher levels of dexterity require not only sufficient muscle strength but also appropriate modulation of grip force, which is essential for successful manual tasks and is influenced by sensorimotor function; impairments in this control contribute to hand dysfunction and may limit independence in ADL [4,7].

In the present case, we focused on the ability to regulate finger force in an individual with CSCI. In addition to muscle strength, we newly evaluated force modulation ability from a motor control perspective, particularly in the extensor carpi radialis muscle, which functions as a wrist extensor and is typically preserved in individuals with C6-level injury [4,8]. By providing visual feedback on force modulation ability and encouraging conscious movement during training and ADL, the individual demonstrated increased frequency of upper-limb use and successfully returned to his previous occupation at discharge, despite no improvement in muscle strength. Written informed consent was obtained from the participant after a full explanation of the study.

## Case presentation

Case description

**Figure 1:**
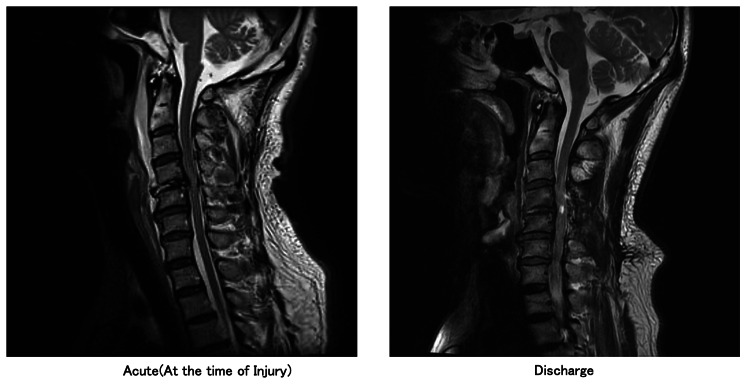
MRI images (Lt: acute, at the time of injury; Rt: discharge).

The patient was a right-handed man in his late 50s. He sustained the injury after slipping and falling forward while mountaineering. A dislocation fracture at the C4/5 level was identified, and posterior fixation surgery was performed. At the time of injury, neurological impairment was classified according to the International Standards for Neurological Classification of Spinal Cord Injury (ISNCSCI), and the patient was graded as AIS B (American Spinal Injury Association Impairment Scale, category B) [8]. The motor score was 10 on the right side (C5: 4, C6: 4, C7: 2, below: 0) and 9 on the left side (C5: 4, C6: 4, C7: 1, below: 0) (Figure 1). The neurological level of injury was C4, and functional independence was evaluated using the Spinal Cord Independence Measure III (SCIM III) [9], with a score of 10, attributable solely to respiration. Before injury, the patient worked as a school teacher. His stated goals were: “I want to be able to take care of myself, even if I need to use a wheelchair,” and “My ultimate goal is to return to work after being discharged home.”

Clinical course: From acute phase to eight months post-injury

Immediately after injury, the occupational therapy program focused on expanding independent ADL performance and included range-of-motion exercises, long-sitting and short-sitting balance training, strengthening exercises for the upper limbs and trunk, and upper-limb functional training. At this stage, eating was possible with the use of a universal cuff once appropriate setup was provided. As the motor score improved over the first two months post-injury, push-up training, scooting movement training, dressing practice, and toileting training were added. At two months post-injury, the AIS grade had improved to D, with a motor score of 34 on the right side and 22 on the left side. The SCIM III score was 22. Subsequently, standing training, floor-to-stand practice, quadruped training, bathing training, and practice of intermittent self-catheterization and spontaneous voiding were conducted. At six months post-injury, the motor score was 44 on the right side and 32 on the left side, and the SCIM III score was 55. A return-to-work program was then initiated. During the training process, the primary therapist visited the patient’s workplace to assess the environment and job requirements. At the second workplace visit, the therapist accompanied the patient to both the workplace and his home to verify whether job-related tasks could be performed using a wheelchair. Job task analysis revealed that finger dexterity was required for tasks such as writing on a blackboard. Because smooth writing with chalk or a pen requires not only maximal muscle strength but also grasp force control through wrist extension, finger function training, fine motor training, and interventions targeting grasp force modulation were subsequently incorporated into the rehabilitation program.

Clinical course: Implementation of force modulation tasks to post-discharge

**Figure 2:**
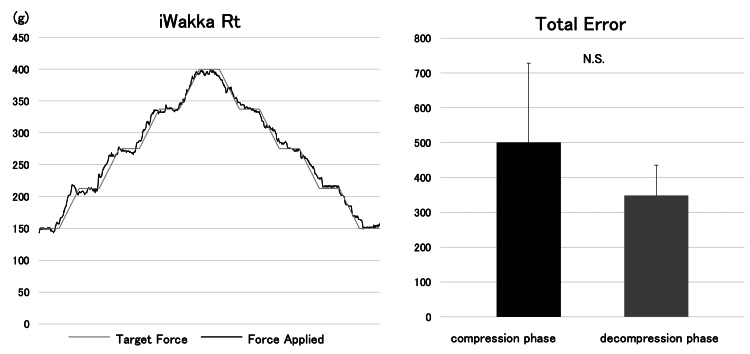
Force modulation task (iWakka). Rt: iWakka; Lt: total error. The graphs were generated using Microsoft Excel (Microsoft Corporation, Redmond, WA).

During the training period, upper-limb rehabilitation was conducted for 60 minutes per day, primarily focusing on muscle strengthening exercises. The patient used a compensatory grasp based on the tenodesis effect to grasp and manipulate objects. To facilitate smoother object grasping and manipulation and to improve finger function with the goal of returning to work, grip force control through force modulation was assessed. Force modulation ability is considered an important component of hand dexterity and plays a critical role in the smooth manipulation of objects [10]. Grip force modulation was evaluated using iWakka (Nagoya Institute of Technology, Nagoya, Japan) [11]. The device consists of a cylindrical grip (height: 80 mm; diameter: 65 mm) divided into two halves and connected by a hinge mechanism, allowing it to open and close. A strain sensor located at the center of the cylinder measures the grip force generated during opening and closing, and the measured force is displayed in real time on an iPad. During the assessment task, the participant was instructed to grasp the device and modulate the applied force to match a target waveform displayed on the iPad screen. The participant attempted to track the target as accurately as possible while maintaining the grasp. Performance during the force modulation task was provided as immediate visual feedback. Grip force modulation performance was analyzed using the error between the target waveform and the generated tracking waveform as the primary outcome measure. The results showed that the error during the force-increasing phase, in which grip force was increased while modulating force, was greater than that during the force-decreasing phase, in which grip force was reduced while modulating force (Figure 2).

**Figure 3:**
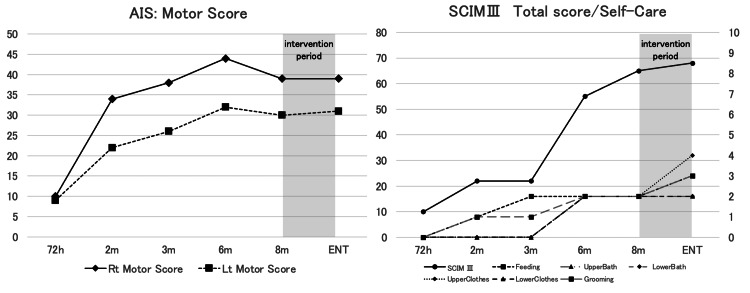
Time course of AIS and SCIM III scores. The graphs were generated using Microsoft Excel (Microsoft Corporation, Redmond, WA). AIS, American Spinal Injury Association Impairment Scale; SCIM III, Spinal Cord Independence Measure III

Based on these findings, the patient was instructed to perform therapeutic exercises and self-directed practice aimed at improving grip force generation using the tenodesis effect and enhancing force modulation through wrist movements. Force modulation training was performed using therapy putty and a ball, in which the patient practiced compressing these objects through wrist movements and gradually releasing the force in a controlled manner. In addition, hand function training was conducted, including writing on a whiteboard with a pen while consciously engaging wrist movements. These interventions were performed daily over a period of three weeks prior to discharge. Subsequently, the patient continued hand function training and practiced writing on a whiteboard using a pen. The patient was eventually discharged from the hospital. At discharge, neurological impairment was classified as AIS D according to ISNCSCI, with motor scores of 39 on the right side and 31 on the left side. The SCIM III score improved to 68 points, with improvements observed in the self-care subdomains of feeding, upper-body dressing, and grooming (Figure 3, Table 1). After discharge, the patient returned to work. The patient reported that during tasks such as writing with a pen, which require controlling grip force while producing writing movements, they became more aware of using wrist palmar flexion and dorsiflexion to modulate grip force while moving the pen.

**Table 1:** Time course of SCIM Ⅲ scores. The table was generated using Microsoft Excel (Microsoft Corporation, Redmond, WA). SCIM Ⅲ, Spinal Cord Independence Measure III

SCIM Ⅲ items		72 h	2 m	3 m	6 m	8 m	ENT
Self-Care							
Feeding		0	1	2	2	2	3
Bathing (upper body)		0	0	0	2	2	2
Bathing (lower body)		0	0	0	2	2	2
Dressing (upper body)		0	0	0	2	2	4
Dressing (lower body)		0	0	0	2	2	2
Grooming		0	1	1	2	2	3
Respiration and Sphincter Management							
Respiration		10	10	10	10	10	10
Bladder		0	0	0	13	13	13
Bowel		0	5	0	5	8	8
Use of toilet		0	1	1	1	4	4
Mobility							
Mobility bed		0	0	2	6	6	6
Transfer bed		0	0	1	2	2	2
Transfer toilet		0	0	1	1	1	1
Mobility indoor		0	2	2	2	2	2
Moderate distance		0	2	2	2	2	2
Mobility outdoor		0	0	0	0	2	1
Stair		0	0	0	0	1	1
Transfer car		0	0	0	1	2	2
Transfer ground		0	0	0	0	0	0
Total score		10	22	22	55	65	68

## Discussion

In this case, force modulation assessment and feedback were introduced as a novel perspective to improve object grasping and manipulation in an individual with CSCI. The intervention contributed to improved upper-limb use during self-care and enabled smooth pen manipulation, ultimately facilitating a successful return to work. Notably, although no improvement was observed in the upper-limb motor score during the period focusing on force modulation training, improvements were observed in SCIM III domains related to self-care, including eating, upper-body dressing, and grooming. These findings suggest that functional independence may be enhanced not only through increased muscle strength but also through motor learning processes that emphasize the regulation of grasping force.

Previous studies have highlighted the importance of force modulation in upper-limb function and object manipulation [4,7,12]. Morita et al. reported improvements in upper-limb function and the Fugl-Meyer Assessment following the implementation of force modulation tasks using the iWakka device in individuals with post-stroke hemiparesis [11]. Similarly, Fukui et al. described finger dexterity as comprising spacing (directional control), timing (temporal control), and grading (force modulation), emphasizing that the ability to modulate force is essential for smooth object manipulation [10]. In rehabilitation for individuals with CSCI, functional training typically focuses on strengthening residual muscles, particularly the extensor carpi radialis longus, to facilitate grasp acquisition using the tenodesis mechanism [13,14]. Increased muscle strength is generally regarded as the primary therapeutic target. However, Johansson et al. reported that the grasp force generated through tenodesis depends on the wrist extension angle and that maximal pinch force is relatively limited, indicating that tenodesis-based grasping primarily functions to hold and stabilize objects [15]. In the present case, despite the absence of measurable changes in muscle strength, the evaluation and feedback of force modulation ability allowed the patient to consciously regulate grasping force during daily activities such as eating and dressing. Furthermore, writing tasks at the workplace require precise adjustment of muscle output while positioning the pen appropriately. Therefore, incorporating interventions that address force modulation was considered beneficial for improving the quality and smoothness of task performance. Interestingly, the results of the force modulation task demonstrated greater error during the compression phase than during the decompression phase. This finding suggests that gradually increasing grasp force through concentric contraction of the extensor carpi radialis longus may be more challenging than gradually decreasing force through eccentric contraction. In contrast, Nakamura reported that in healthy adults, force modulation errors during pinch tasks were greater during the decompression phase than during the compression phase [16]. These differences may reflect variations in motor control strategies between individuals with CSCI using tenodesis-based grasping and healthy individuals. In individuals with CSCI, impaired finger coordination and reduced neuromuscular control may contribute to difficulties in regulating grasp force [17]. Because this compensatory grasping strategy differs from that of healthy adults in terms of muscle activation patterns, distinct force modulation strategies may emerge. The present findings suggest that individuals with CSCI may need to acquire alternative motor control strategies for effective object manipulation in daily activities. However, this study has several limitations. As this report describes a single case, the generalizability of the findings is limited. In addition, quantitative changes in motor control strategies were not directly measured beyond the force modulation task. Future studies involving larger samples and detailed biomechanical analyses are needed to further clarify the role of force modulation in upper-limb rehabilitation for individuals with CSCI. Taken together, the present findings highlight the potential importance of incorporating force modulation assessment and feedback into rehabilitation programs to facilitate efficient and functional hand use in daily activities.

## Conclusions

In upper-limb rehabilitation for individuals with CSCI aimed at achieving ADL independence and improving quality of life through tenodesis-based grasping, force modulation ability was evaluated, and its characteristics were fed back to the patient as a new perspective in addition to strengthening residual muscles. As a result, improvements in SCIM III were observed despite no change in the AIS motor score. Furthermore, the intervention contributed to the acquisition of smooth pen-writing ability required for return to work. These findings suggest that interventions focusing on force modulation ability, in addition to muscle strengthening, may be effective for individuals with CSCI.

## References

[1] Simpson LA, Eng JJ, Hsieh JT, Wolfe DL (2012). The health and life priorities of individuals with spinal cord injury: a systematic review. J Neurotrauma.

[2] Anderson KD (2004). Targeting recovery: priorities of the spinal cord-injured population. J Neurotrauma.

[3] Lu X, Battistuzzo CR, Zoghi M, Galea MP (2015). Effects of training on upper limb function after cervical spinal cord injury: a systematic review. Clin Rehabil.

[4] Jimbo K, Miyata K, Yuine H (2024). Classification of upper-limb dysfunction severity and prediction of independence in activities of daily living after cervical spinal-cord injury. Spinal Cord.

[5] Lei Y, Perez MA (2018). Phase-dependent deficits during reach-to-grasp after human spinal cord injury. J Neurophysiol.

[6] Harvey L (2008). Management of Spinal Cord Injuries A Guide for Physiotherapists. ELSEVIER.

[7] Davidson S, Learman K, Rosenfeldt AB, Zimmerman E, Alberts JL (2019). Parkinson’s disease impairs grip force release during a sinusoidal force tracking task. Exp Brain Res.

[8] Kirshblum SC, Burns SP, Biering-Sorensen F (2011). International standards for neurological classification of spinal cord injury (revised 2011). J Spinal Cord Med.

[9] Catz A, Itzkovich M, Tesio L (2007). A multicenter international study on the Spinal Cord Independence Measure, version III: Rasch psychometric validation. Spinal Cord.

[10] Fukui T, Inoue K, Tunehisa K (2020). Analysis of three basic elements in hand dexterity: inspection of its speed changes. Kawasaki J Med Welfare.

[11] Morita Y, Ando K, Nomura M (2019). Verification of usefulness of testing/training device of adjustability for grasping force on hemiplegic patients after stroke. Trans Soc Instrum Control Eng.

[12] Johansson RS, Westling G (1984). Roles of glabrous skin receptors and sensorimotor memory in automatic control of precision grip when lifting rougher or more slippery objects. Exp Brain Res.

[13] Jung HY, Lee J, Shin HI (2018). The natural course of passive tenodesis grip in individuals with spinal cord injury with preserved wrist extension power but paralyzed fingers and thumbs. Spinal Cord.

[14] Harvey L (1996). Principles of conservative management for a non-orthotic tenodesis grip in tetraplegics. J Hand Ther.

[15] Johanson ME, Murray WM (2002). The unoperated hand: the role of passive forces in hand function after tetraplegia. Hand Clinics.

[16] Nakamura M, Hirota M, Nakamura M (2013). Characteristics of adjusting precision grip strength: relationship between coordination and pulp-to-pulp distance when applying and reducing pinch pressure. Japan J Occup Ther.

[17] Latash ML, Scholz JF, Danion F, Schöner G (2002). Finger coordination during discrete and oscillatory force production tasks. Exp Brain Res.

